# Conjunctival administration of S+ketamine in rabbits

**DOI:** 10.1186/cc12327

**Published:** 2013-03-19

**Authors:** L Hess, J Malek, A Kurzova, Z Simunkova, M Votava

**Affiliations:** 1 Institute for Clinical and Experimental Medicine, Prague, Czech Republic; 2 3rd Medical Faculty of Charles University, Prague, Czech Republic

## Introduction

Delivering analgesics via conjunctival application could provide rapid and convenient pain relief in disaster medicine. There are sporadic reports from the USA concerning inhalation administration of aerosol with various drugs producing a wide variety of effects from anxiolysis, sedation, and loss of aggressiveness to immobilisation. We attempted to determine in an animal experiment whether conjunctival administration of S+ketamine could produce significant effect without side effects.

## Methods

After ethic committee approval, 10 rabbits were administered conjunctival S+ketamine 2.5 mg/kg. Measured parameters were SpO_2_, blood pressure (BP) and heart rate (HR) before administration and in 1-minute intervals and immobilisation time (loss of righting reflex). The measurements were performed for 20 minutes. Conjunctival irritation was measured 1, 10 and 20 minutes after administration according to modified technical standard EN IS O 10993-10. ANOVA test was used for statistical analysis of hemodynamic parameters.

## Results

The immobilisation time was 207 ± 60 seconds. There were no changes in cardiorespiratory parameters (initial, 10 and 20 minutes after administration: HR 255.5 ± 24.7, 265.8 ± 26.0 and 267.8 ± 20.4, systolic BP 114.3 ± 8.6, 108.2 ± 10.7 and 113.9 ± 11.9 mmHg, diastolic BP 66.8 ± 11.6, 68.4 ± 10.6 and 69.7 ± 9.3 mmHg and SpO_2 _99.2 ± 1.0, 98.2 ± 0.6 and 98.7 ± 0.5). These results are in contrast to conjunctival administration of sufentanil 5 μg/kg that caused a significant decrease of SpO_2 _and HR [1]. We can speculate that the reason for stability of cardiorespiratory parameters was due to the sympathoadrenergic effect of ketamine or due to the method of administration. There were no signs of conjunctival irritation in any animal (S+ketamine is a preservative-free solution).

## Conclusion

Conjunctival S+ketamine 2.5 mg/kg in rabbits produced rapid onset without changes in cardiorespiratory parameters and without signs of irritation of the eye. The results of our project warrant further research to increase the variety of drugs and methods of their administration for anxiolysis, sedation and analgesia in disaster medicine.
